# Giant Cell Tumor of the Distal Humerus: A Rare Location With a Good Response to Denosumab

**DOI:** 10.7759/cureus.114363

**Published:** 2026-08-11

**Authors:** Mahy Mohamed El Kerim, Manal Azizi, Mohamed Belahcen, Amal Bennani, Imane Kamaoui, Ayad Ghanam, Maria Rkain

**Affiliations:** 1 Pediatrics (Residency), Mohammed VI University Hospital, Oujda, MAR; 2 Pediatrics, Faculty of Medicine and Pharmacy, Mohammed VI University Hospital, Mohammed 1st University, Oujda, MAR; 3 Pediatric Surgery, Faculty of Medicine and Pharmacy, Mohammed VI University Hospital, Mohammed 1st University, Oujda, MAR; 4 Anatomopathology, Faculty of Medicine and Pharmacy, Mohammed VI University Hospital, Mohammed 1st University, Oujda, MAR; 5 Oncoradiology, Faculty of Medicine and Pharmacy, Mohammed VI University Hospital, Mohammed 1st University, Oujda, MAR

**Keywords:** denosumab, distal humerus, giant cell tumor of bone, pediatrics, rankl inhibition, skeletally immature

## Abstract

Giant cell tumors (GCTs) are rare, benign, locally aggressive neoplasms. They typically affect the metaphyseal region; their occurrence at the distal humerus is rare, making their management challenging.

We report the case of a 13-year-old girl, skeletally immature, admitted for a painful, progressive mass at her right elbow (circumference 26 cm) with functional limitation. Radiological evaluation by magnetic resonance imaging (MRI revealed a large osteolytic epiphysometaphyseal lesion of the right distal humerus, centered on the medial epicondyle and measuring 36 × 35 × 60 mm, with cortical rupture and soft-tissue invasion (Campanacci grade III). The bone biopsy revealed a GCT with no signs of malignancy. The staging workup was negative. Given the complex joint location and the patient’s young age, the multidisciplinary team decided to initiate anti-receptor activator of nuclear factor-κB ligand (RANKL) therapy with denosumab (70 mg/m² subcutaneously (SC)) on days 1, 8, 15, and 28, followed by monthly administration, along with calcium and vitamin D supplementation. The seven-month follow-up was favorable: regression of the swelling (circumference 20 cm), complete joint release, and lesion stability on the follow-up MRI, with no calcium-phosphate complications.

Denosumab is used in situations where surgery is not an option to reduce tumor size and control its progression. Its use increases the risk of calcium-phosphorus abnormalities and growth disorders. Our case demonstrated that a well-defined treatment regimen combined with calcium-phosphorus supplementation could control the aggressiveness of GCTs.

## Introduction

Giant cell tumor (GCT) of bone is a rare, intermediate-grade, benign yet locally aggressive, osteolytic bone neoplasm [1]. It typically affects the metaphyseal region of long bones, primarily the distal femur and proximal tibia, in individuals aged 20 to 45 years, with a predominance in women [2,3]. A tumor in the distal humerus is rare. Treatment is primarily surgical; however, denosumab therapy is indicated in complex cases. The use of denosumab in skeletally immature patients remains a subject of ongoing debate. As a receptor activator of nuclear factor-κB ligand (RANKL) inhibitor, denosumab theoretically carries the risk of interfering with normal bone remodeling and growth plate physiology, potentially affecting longitudinal bone growth. Furthermore, the optimal dosing regimen, treatment duration, and long-term safety profile in the pediatric population have not yet been established through prospective controlled studies. Reported concerns also include the risk of osteoclastic rebound hypercalcemia and local tumor recurrence following treatment discontinuation [4,5].

We report the case of a 13-year-old girl presenting with a GCT in a rare location (the medial epicondyle of the distal humerus) who has shown a favorable long-term response to denosumab.

## Case presentation

**Figure 1:**
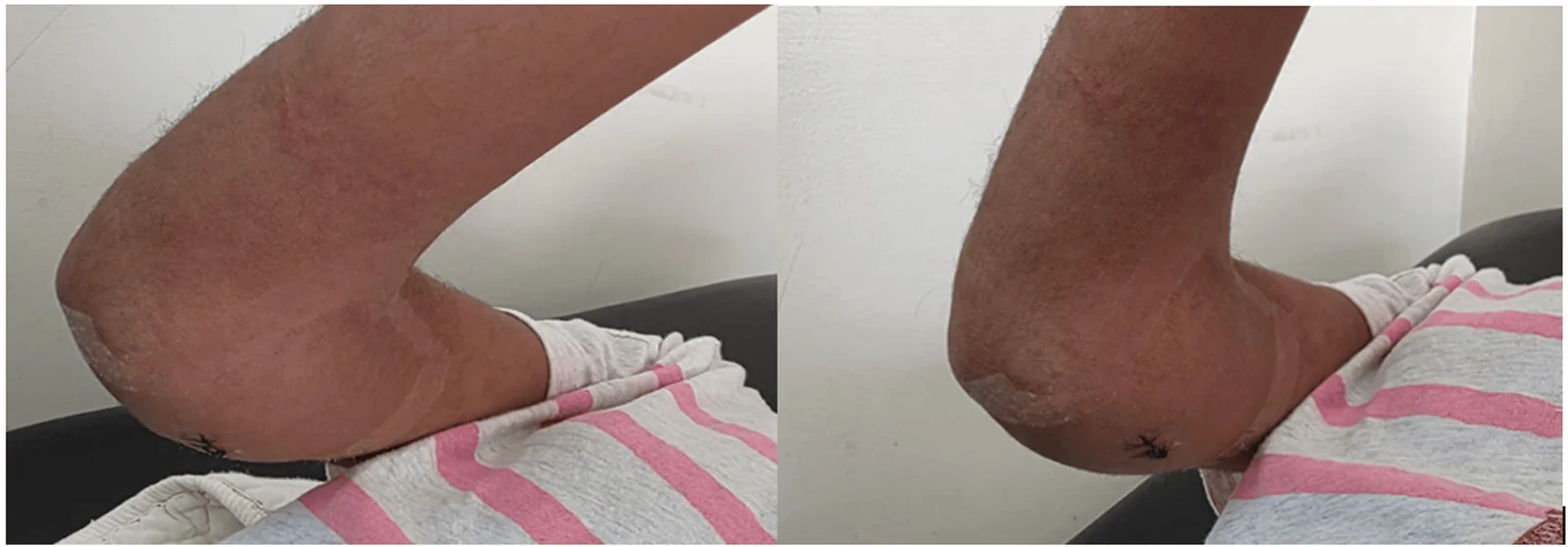
Clinical photographs showing a posterior-medial swelling of the right elbow at the level of the medial epicondyle of the distal humerus, with a visible biopsy scar on the medial aspect.

This was a 13-year-old girl born to non-consanguineous parents, with no family history of cancer or degenerative bone disease. Her medical history was notable for trauma to the right upper limb six months before admission. She presented with a two-month history of swelling of the right elbow that had gradually increased in size. The swelling was painful on palpation and associated with functional impairment, without signs of local inflammation or neurovascular compression. On clinical examination, the girl was conscious and in good general condition, without pain, presenting a red, swollen right elbow with a circumference of 26 cm. The limb was flexed at 90°, with limited passive and active range of motion; extension was limited by pain, with no associated sensory-motor deficits or vascular signs (Figures 1a, 1b).

Laboratory results

Inflammatory markers were negative, and calcium and phosphorus levels were normal.

Radiological findings

**Figure 2:**
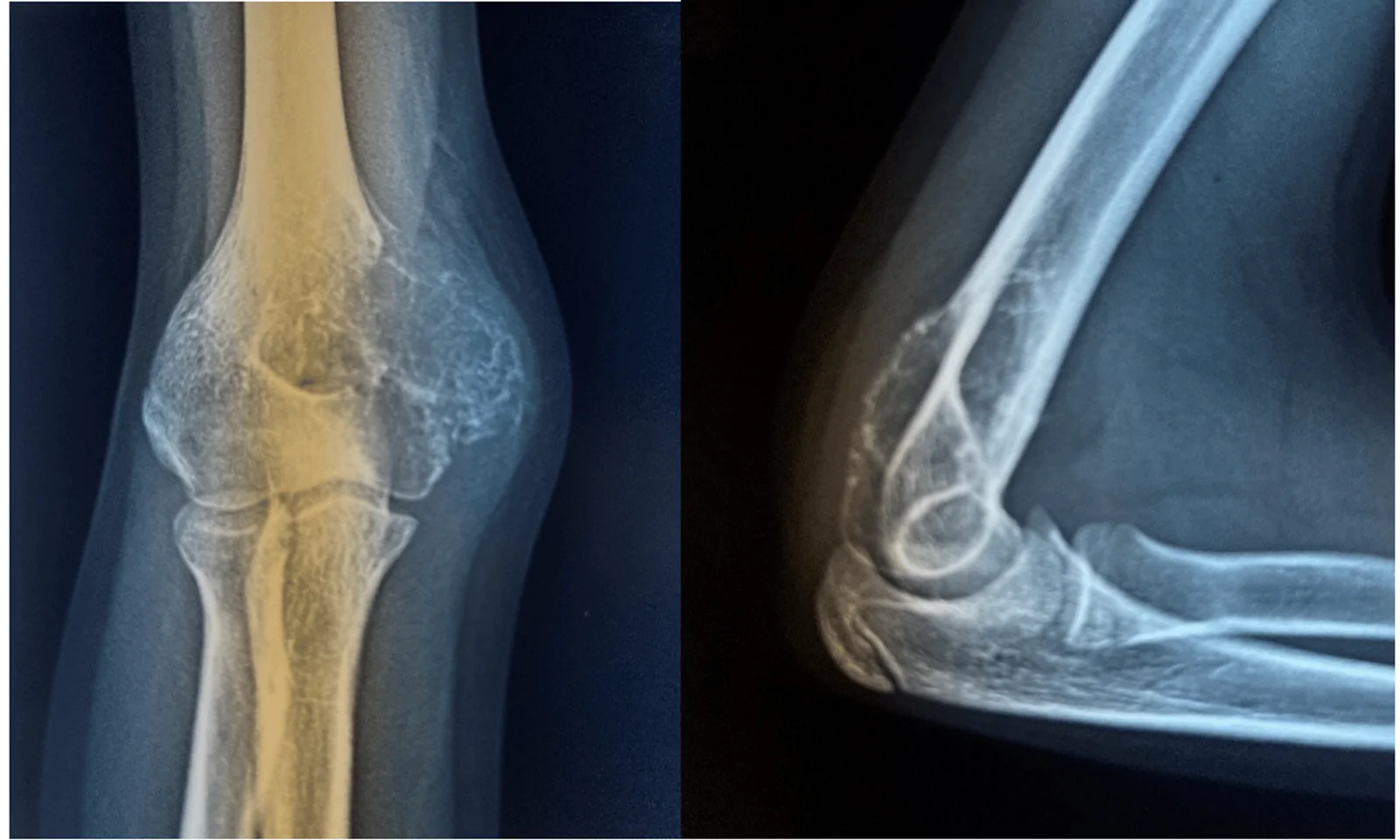
Anteroposterior and lateral plain radiographs of the right elbow demonstrating a large osteolytic lesion centered on the medial epicondyle of the distal humerus, with an associated periosteal reaction and cortical thinning.

**Figure 3:**
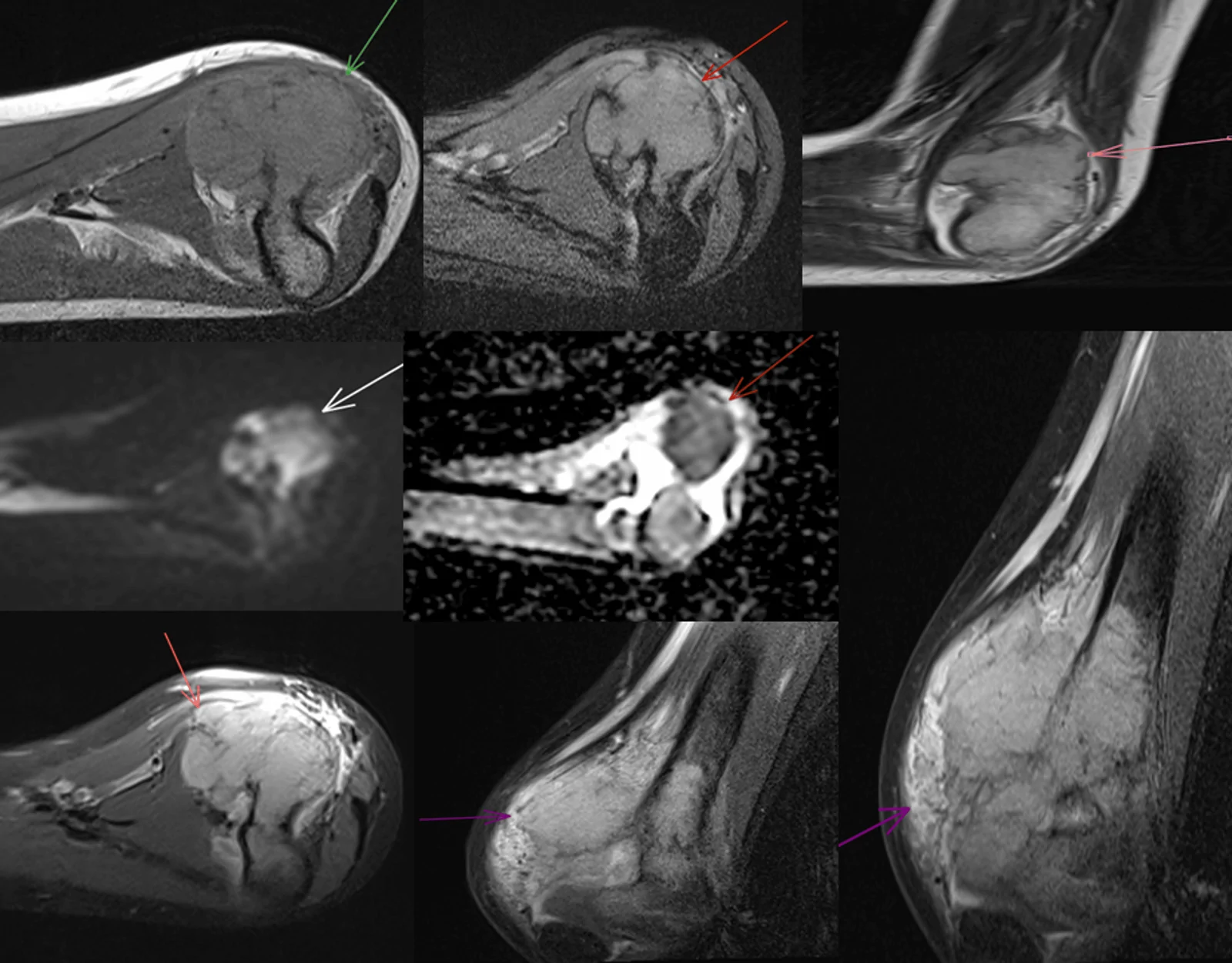
MRI images in axial, coronal, and sagittal planes: showing an aggressive epiphyseal-metaphyseal lesion of the right distal humerus centered on the medial epicondyle, appearing as a mild T1 hyper-intensity (green arrow), an intermediate T2 hyper-intensity (pink and red arrows), and diffusion restriction (white and brown arrows), with enhancement following gadolinium injection (purple and orange arrows).

Anteroposterior and lateral X-rays of the elbow show an osteolytic lesion on the medial aspect of the elbow opposite the medial epicondyle, with a periosteal reaction (Figures 2a, 2b). MRI of the elbow in axial, coronal, and sagittal planes revealed an aggressive epiphyseal-metaphyseal lesion of the right distal humerus centered on the medial epicondyle, measuring 36 × 35 × 60 mm (Figure 3). A thoraco-abdominal-pelvic CT scan showed no metastatic lesions.

Histological findings

**Figure 4:**
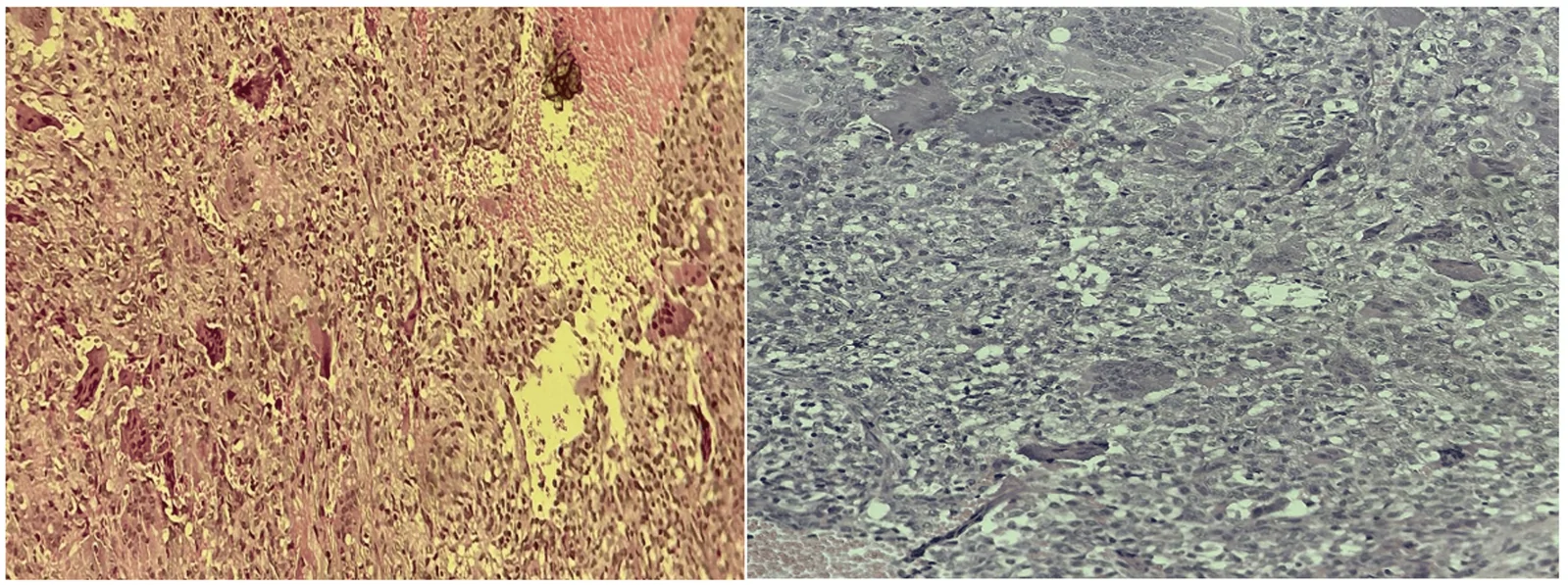
(a) Histological image showing a tumor proliferation with a diffuse architecture composed of two cell types: multinucleated cells and mononucleated cells (HES, x10); (b) the mononucleated cells are globular and monomorphic, and the giant cells have ovoid nuclei without cytoplasmic atypia (HES, x40) HES, hematoxylin-eosin-saffron

The pathological examination revealed a histological appearance consistent with a GCT without signs of malignancy (Figure 4).

**Figure 5:**
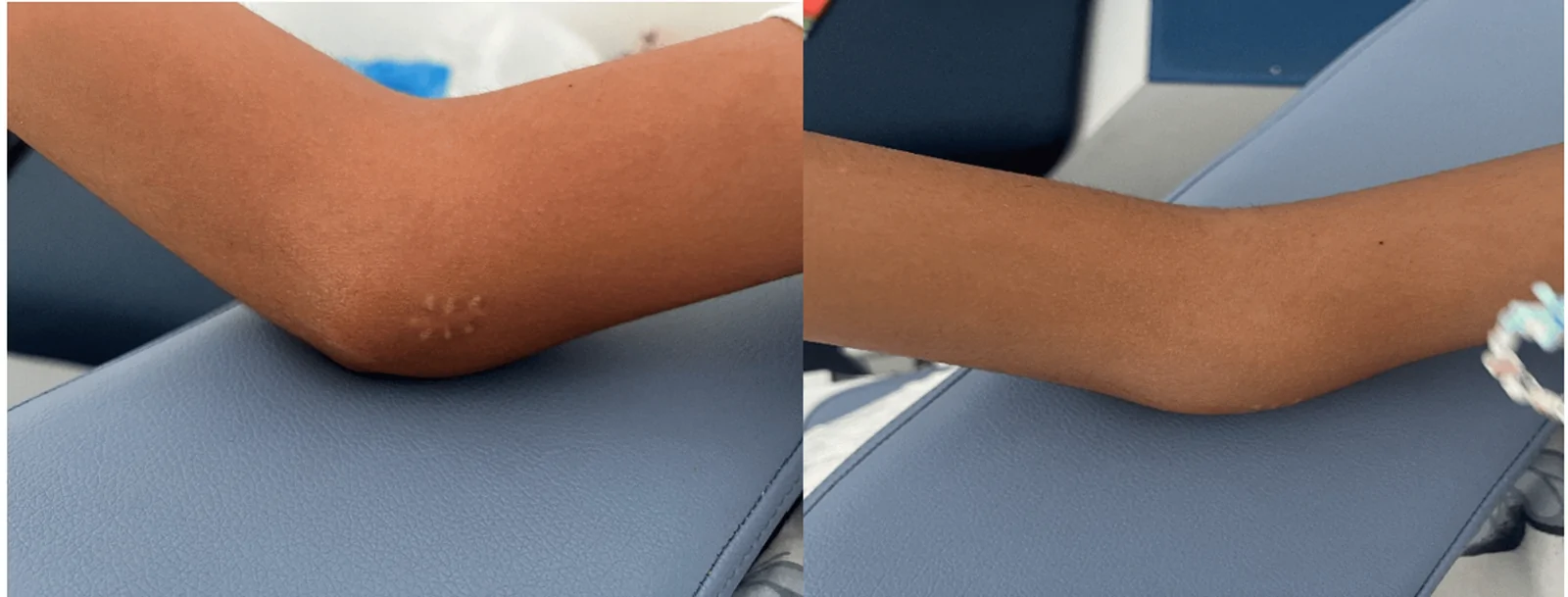
Clinical photographs taken after seven months of denosumab therapy, demonstrating significant reduction in swelling at the medial epicondyle of the distal humerus and restoration of elbow range of motion up to 150° of flexion.

**Figure 6:**
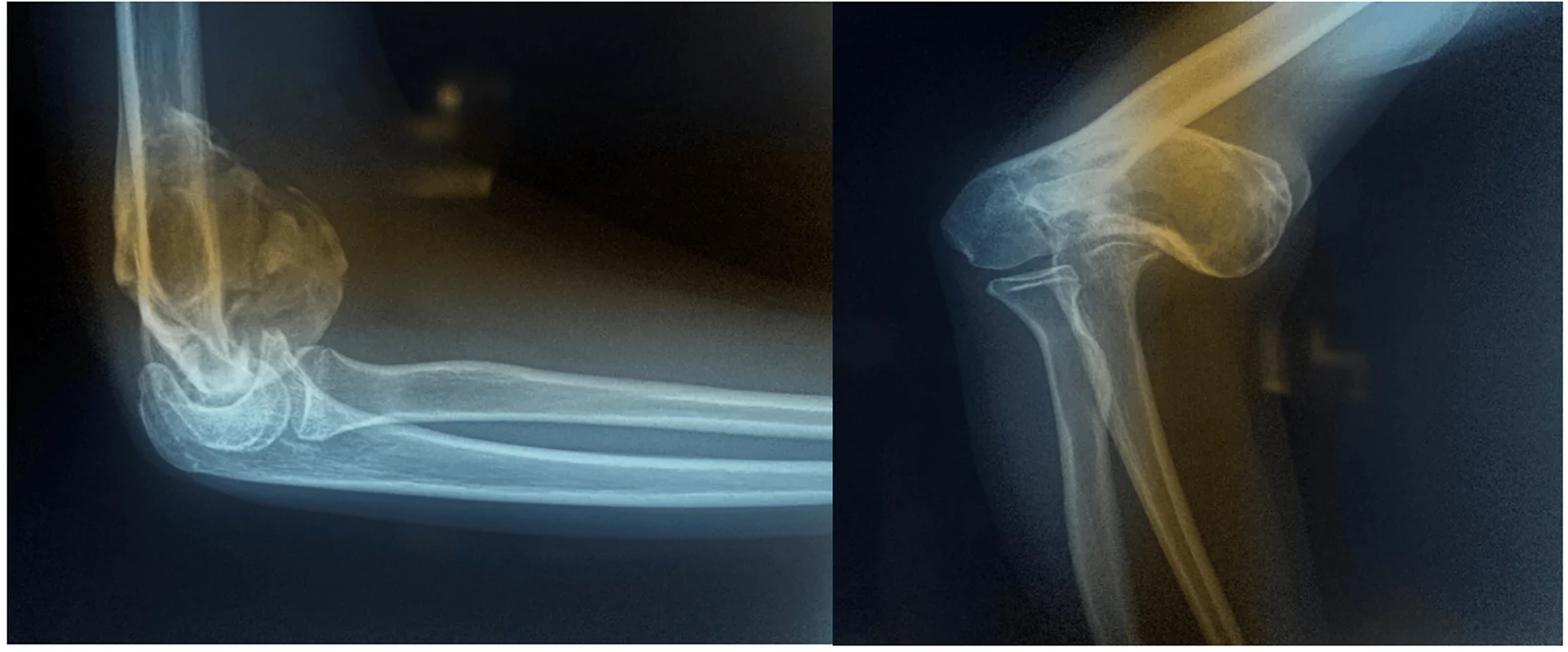
Anteroposterior and lateral plain radiographs of the right elbow after six months of denosumab therapy, showing marked regression of the osteolytic lesion at the medial epicondyle of the distal humerus, with progressive remineralization of the tumor matrix, consolidation, and formation of a peripheral neo-cortical shell, consistent with a favorable radiological response to anti-RANKL therapy RANKL, receptor activator of nuclear factor-κB ligand

Given the tumor’s location, the multidisciplinary team decided to treat the patient with denosumab 70 mg/m² on days 1, 8, 15, and 28, followed by monthly doses. The patient’s condition improved, as the tumor shrank and the elbow regained mobility and became pain-free (Figure 5). The circumference decreased to 20 cm, with regression of the osteolytic lesion on the X-ray and improved ossification (Figure 6). The treatment was tolerated; the patient experienced no treatment-related complications and has had normal calcium and phosphorus levels since the start of treatment.

## Discussion

Giant cell bone tumor is a rare, locally aggressive, osteolytic benign bone tumor first described by Cooper and Travers in 1818 [3]. It most commonly affects the epiphyses of long bones in people aged 20 to 45 years, with a predominance in females, and is rare in children before the growth plates have closed [4,5]. The incidence among children under 19 years of age has been reported in several studies and ranges from 1.7% to 10.6% of all patients with GCT [6]. The most commonly affected sites are the proximal tibia and the distal femur; however, less than 1% of cases involve the distal radius and the sacrum [2,7]. Lesions of the humerus account for approximately 6% of all cases, but nearly all of them are located in the proximal humerus, with lesions of the distal humerus being extremely rare [8,9]. The present case illustrates a rare manifestation of a GCT in the distal humerus in a 13-year-old girl.

In general, the clinical presentation often involves a gradually progressive mass, as in the case of our patient. Laboratory tests are often normal; however, a calcium-phosphate panel is ordered to rule out hyperparathyroidism. Radiologically, a standard X-ray helps guide the diagnosis by showing a purely osteolytic or honeycomb-like pattern, with or without a periosteal reaction or cortical fracture. Computed tomography (CT) and magnetic resonance imaging (MRI) help further guide the diagnosis by assessing for soft-tissue invasion and ruling out differential diagnoses, particularly chondroblastoma, aneurysmal cysts, or epiphyseal locations of clear-cell chondrosarcomas. In our patient, a standard X-ray of the elbow revealed an osteolytic lesion on the medial aspect of the elbow opposite the medial epicondyle with a periosteal reaction. MRI of the elbow showed an aggressive epiphyseal-metaphyseal lesion of the right distal humerus centered on the medial epicondyle, classifying the lesion as Campanacci grade III [10,11]. Histologically, the GCT has a crumbly appearance and a color ranging from dark brown to reddish. The histological appearance is traditionally characterized by a stroma composed primarily of mononuclear spindle-shaped cells and monocytes in the foreground, with several large, multinucleated giant cells in the background. Neoplastic cells (osteoclast precursors and spindle-shaped stromal cells) and reactive cells, such as large, multinucleated giant cells resembling osteoclasts, constitute the GCT [12-14].

About treatment, GCTs are locally aggressive tumors. The choice of treatment depends on the tumor’s location, invasion of the soft tissues, and proximity to the joint. Treatment is often surgical, either intralesional, with simple curettage; conventional curettage followed by bone grafting; or curettage and filling with adjuvants (liquid nitrogen, distilled water, phenol, or surgical cement); or extralesional, with marginal resection and reconstruction, wide resection, or amputation [15,16]. However, other alternatives are possible: radiation therapy, cryotherapy, chemotherapy, and biologic therapy, notably denosumab.

Denosumab is a fully human monoclonal antibody that specifically inhibits RANKL, thereby preventing osteoclast-mediated bone resorption [17,18]. Its subcutaneous administration results in rapid and sustained suppression of bone turnover in patients with multiple myeloma and osteolytic bone disease, as well as in patients with breast or prostate cancer with bone metastases. RANKL inhibition by denosumab in patients with GCT could slow bone destruction and eliminate giant cells. It is indicated for inoperable GCTs, those for which surgery would be morbid, and metastatic forms [19]. The clinical relevance of denosumab’s role was established based on the results of two phase studies published by Thomas and Chawla in 2010 and 2013, respectively [20,21]. The dosage of denosumab in pediatric patients ranges from the adult dose of 120 mg/m² administered subcutaneously (for patients weighing more than 50 kg) to the minimum dose of 70 mg/m² (for patients weighing less than 50 kg), which has already been described in certain situations, such as aneurysmal cysts, to avoid adverse effects on growth [22,23]. Vitamin D and calcium supplementation are necessary, along with monitoring of calcium and phosphorus levels.

Our patient had an aggressive epiphyseal-metaphyseal lesion of the right distal humerus, requiring complex surgery that jeopardizes the limb’s prognosis. The decision made during the multidisciplinary team meeting was to treat the patient with subcutaneous denosumab at a dose of 70 mg/m² on days 1, 8, 15, and 28, followed by monthly injections, along with vitamin D supplementation at 1,000 IU/day and calcium supplementation at 1,000 mg/day. The course of the disease was favorable, with restoration of elbow mobility following the induction phase and a reduction in circumference to 20 cm after seven months of treatment. Radiological follow-up via standard X-ray showed regression of the osteolytic lesion, and monitoring of calcium and phosphorus levels was normal, with a lower serum calcium level of 79 mg/L and no signs of clinical hypocalcemia. Given our patient’s tolerance of denosumab, we plan to continue treatment for 18 months with quarterly radiological follow-up.

## Conclusions

GCTs are locally aggressive tumors, and their treatment is often controversial, with surgery being the treatment of choice. Since the discovery of denosumab, treatment options have expanded. It has made it possible to avoid high-risk surgeries while providing better tumor control; however, the optimal duration of treatment and the interval between doses are still subjects of debate and are currently determined on a case-by-case basis, according to recent reports in the literature. This case illustrates that denosumab may be effective as a neoadjuvant treatment or for stabilizing lesions in children with inoperable GCTs. Good compliance and tolerability were observed, even with prolonged treatment. No adverse effects were observed. A more in-depth analysis of adverse effects following treatment discontinuation is needed, particularly regarding the resumption of osteoclastic activity. These results are preliminary, and further studies are needed with a larger number of cases and a longer follow-up period.
